# Enzyme replacement therapy reduces the risk for wheelchair dependency in adult Pompe patients

**DOI:** 10.1186/s13023-018-0824-4

**Published:** 2018-05-22

**Authors:** Jan C. van der Meijden, Michelle E. Kruijshaar, Dimitris Rizopoulos, Pieter A. van Doorn, Nadine A. M. E. van der Beek, Ans T. van der Ploeg

**Affiliations:** 1 Center for Lysosomal and Metabolic Diseases, Erasmus MC University Medical Center, PO Box 2040, 3000 CA Rotterdam, the Netherlands; 2 Department of Biostatistics, Erasmus MC University Medical Center, Rotterdam, the Netherlands; 3 Department of Neurology, Erasmus MC University Medical Center, Rotterdam, the Netherlands

**Keywords:** Pompe disease, Enzyme replacement therapy, ERT, Wheelchair, Respiratory support, Hazard ratio

## Abstract

**Background:**

Pompe disease is a rare metabolic myopathy. In adult patients, progressive weakness of limb-girdle and respiratory muscles often leads to wheelchair and respirator dependency. Clinical studies have shown enzyme replacement therapy (ERT) to positively affect motor and respiratory outcomes. Here we investigate whether ERT reduces patients’ risk of needing a wheelchair or respirator.

**Methods:**

Data were collected as part of a prospective international survey, the IPA/Erasmus MC Pompe survey, which was conducted annually between 2002 and 2016. We excluded patients who were already using a wheelchair or respirator, those under 18 at survey entry, and those who had missing information. Time-dependent Cox proportional hazard models were used.

**Results:**

The inclusion criteria for analyzing the risk of wheelchair use were met by 189 patients (median age 47 years; range 18–75). During follow-up, 126 (67%) started ERT. Over 1120 person-years of follow-up (median 5 years), 46 became wheelchair dependent, 16 of whom used ERT. After adjustment for disease duration, sex and country, ERT reduced the risk for wheelchair use (HR 0.36; 95% CI 0.17–0.75). For analyses of respirator use, 177 patients met the inclusion criteria (median age 46 years; range 18–73). Over 1190 person-years of follow-up (median 6 years), 125 patients (71%) were treated and 48 started respiratory support, 28 of whom received ERT. We found no association between ERT and the risk for respirator use (HR 1.23; 95% CI 0.61–2.47).

**Conclusions:**

Our study found that ERT reduced the risk for wheelchair dependency. We could not demonstrate an effect on respiratory support.

## Background

Pompe disease is an autosomal recessive metabolic myopathy for which enzyme replacement therapy (ERT) with alglucosidase alfa has been available since 2006 [1, 2]. In adults, the disease is characterized by progressive limb-girdle and respiratory muscle weakness. In most cases, this ultimately leads to the use of a wheelchair and/or respiratory support [3, 4]. Wheelchair dependency and the use of respiratory support considerably impact a patient’s ability to participate in daily life activities, and reduce quality of life [5, 6]. It should therefore be an important treatment goal to prevent the disease from progressing to the point that a patient becomes dependent on these aids.

Many studies have evaluated the effects of ERT in adult patients with Pompe disease. While these have shown that ERT has a positive effect on motor function and/or lung function [7–15], its effect on wheelchair dependency and respiratory support has been reported only in a few cases.

Together with the International Pompe Association (IPA), our center has systematically collected data on patients with Pompe disease since 2002, well before the approval of ERT. This IPA/Erasmus MC Pompe survey has consistently followed a large international cohort of patients over many years, both before and during ERT. Earlier findings from the survey include the demonstration of a positive effect of ERT on survival [16]. Using data from this survey, we investigated whether ERT reduces the risk that a patient will need a wheelchair or respiratory support.

## Methods

### Patients

A detailed description of the survey’s design has been published previously [16]. Since 2002, patients have been recruited through national patient organizations in Canada, Germany, France, the Netherlands, the United Kingdom and the United States. Recruitment was independent of patients’ disease severity. All patients have provided informed consent.

Annual survey questions included items on the use of a wheelchair and respiratory support. Disease duration was calculated as the number of years since diagnosis. In our analyses we included all questionnaires completed before July 2016.

The current study included patients aged 18 years and above at inclusion in the survey. Patients who already used a wheelchair or respiratory support at survey entry (i.e. who had already had the “event”) were excluded from the analyses, as were those who had completed the survey only once (no follow-up) or had incomplete information on the events or disease duration.

### Statistical analysis

Time-dependent Cox proportional hazard models were used to calculate the effect of ERT on the risk of using a wheelchair or respiratory support. Models were developed separately for both outcomes.

Age was used as the time scale of the analysis, each patient being followed from the age at inclusion in the survey, until the date of last follow-up (censoring), or until becoming wheelchair or ventilator dependent. ERT was assessed as a time-dependent variable that switched from 0 to 1 when patients started treatment. This approach allowed patients’ to contribute both treated and untreated person-years of follow-up to the analyses. The following covariates were chosen a priori: disease duration, gender and country of residence. Like ERT, disease duration was included as a time-dependent covariate, updating when patients started treatment.

Results are presented as hazard ratios (HRs) with 95% confidence intervals (CIs). The proportional hazards assumption was checked by plotting scaled Shoenfeld residuals and correlating them with the Kaplan Meier estimate of the survival function.

Since both analyses originate from the same population, we used the Holm method to correct for multiple testing [17]. Please note that the two populations overlap, but are not entirely the same since some of the exclusions depend on whether one is assessing wheelchair or ventilator dependency.

Statistical tests were performed using R version 3.3.1 including the survival package [18, 19]. A *p*-value < 0.05 was considered significant.

## Results

**Fig. 1:**
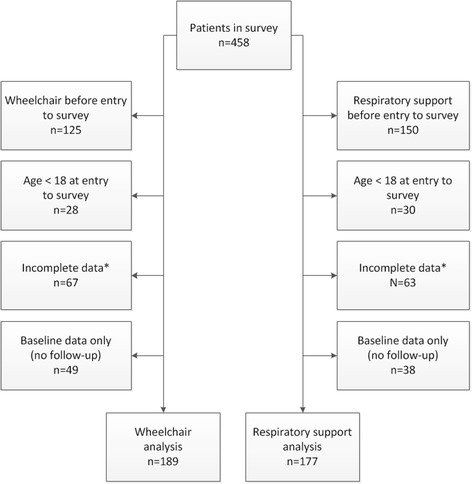
Inclusion flowchart. *Data was incomplete if either the age at which the event occurred or the disease duration was unknown

Overall, 458 patients participated in the IPA/Erasmus MC Pompe survey between 2002 and July 2016. The inclusion flow-chart in Fig. 1 shows that 189 patients were eligible for analysis of the effect of ERT on wheelchair dependency and that 177 were eligible for analysis of respiratory support (131 patients were in both analyses). 125 patients (27%) were excluded as they already used a wheelchair at survey entry; 150 (33%) were excluded because they already required respiratory support.

Table 1 compares the baseline characteristics of patients included in the two analyses with those of all patients participating in the IPA/Erasmus MC survey. Overall, patients who participated in the survey (*n* = 458) were equally distributed between the sexes, and entered the survey at a median age of 47 years. Seventy percent started ERT at some point during their follow-up. Patients who fulfilled the inclusion criteria for our analyses had a shorter disease duration compared to those who were excluded from the analyses (both *p* < 0.001). This might be explained by the fact that patients who required a wheelchair or ventilator at survey entry had to be excluded from the analysis. Patients included in the analyses were also more frequently from the Netherlands (both *p* < 0.001). Patients in the ventilator analysis were more frequently female than those excluded from this analysis (*p* < 0.001).

**Table 1:** Characteristics of patients participating in the analysis of the effect of ERT on the use of a wheelchair and respiratory support, and of all patients included in the IPA/Erasmus MC Pompe survey

	Included in analyses		All participants Survey (*n* = 458)
	Risk of using a wheelchair (*n* = 189)	Risk of using respiratory support (*n* = 177)	
Female, *n* (%)	98 (52%)	109 (62%)*	230 (50%)
Median age at entry into the survey, years (range)	47 (18–75)	46 (18–73)	47 (2–81)
Median age at diagnosis, years (range)	39 (3–72)	39 (0–72)	38 (0–72)
Median disease duration at entry, years (range)	5 (0–39)*	5 (0–31)*	7 (0–39)
Country of residence, *n* (%)	*	*	
Netherlands	78 (41%)	87 (49%)	134 (29%)
United Kingdom	9 (5%)	9 (5%)	36 (8%)
United States	44 (23%)	36 (20%)	124 (27%)
Germany	26 (14%)	25 (14%)	66 (14%)
Other	32 (17%)	20 (11%)	98 (21%)
ERT^a^, *n* (%)	126 (67%)	125 (71%)	319 (70%)
Median age start ERT, years (range)	48 (13–77)	49 (13–74)	47 (3–77)
Median follow-up duration, years (range)	5 (1–14)	6 (1–14)	N.A.
Events during follow-up, *n* (%)	46 (24%)	48 (27%)	N.A.
Median age at event, years (range)	52 (21–76)	50 (24–73)	N.A.

^a^most patients started ERT at some point during their follow-up; 57 patients in the analyses of wheelchair use and 37 in the analyses of use of respiratory support were already receiving ERT at survey entry. *: the median/proportion was significantly different (*p* < 0.05) from the median/proportion in the patients who were excluded from this analysis

Table 2 shows the results of the time-dependent Cox proportional hazard regression models. A total of 1120 person-years of follow-up were available for our analysis of the effect of ERT on wheelchair use (median follow-up 5 years, Table 1). Sixteen events occurred over 652 treated-person years and 30 events over 468 untreated-person years. After adjustment for disease duration at survey entry and at start of ERT, and also for sex and country, ERT significantly reduced the risk of becoming wheelchair dependent (hazard ratio of 0.36; CI 0.17–0.75, Table 2). In other words, at any point during follow-up, a treated patient had a 64% lower risk for becoming wheelchair dependent than an untreated patient. Disease duration was an important predictor of becoming wheelchair dependent: the risk for becoming wheelchair dependent was over three times higher in those whose disease duration was over 10 years than in those with less than 5 years. Country of residence was also significantly associated with the risk for wheelchair use, with patients from countries other than the Netherlands, Germany, and the US having a higher risk of using a wheelchair than those from the Netherlands.

**Table 2:** Multivariate time-dependent Cox regression analysis of wheelchair use (A) and the need for respiratory support (B)

	Events	Person-years	HR	95% CI	*p*-value
A. Wheelchair use					
Treatment^a^					
Untreated (ref)	30	468			
ERT	16	652	0.36	(0.17–0.75)	*0.004*
Disease duration^a^					
< 5 years (ref)	10	464			
5–10 years	10	332	1.19	(0.41–3.48)	1
> 10 years	26	324	3.87	(1.55–9.61)	*0.002*
Sex					
Male (ref)	17	583			
Female	29	537	1.80	(0.88–3.69)	0.13
Country of residence					
Netherlands (ref)	13	531			
Germany	9	173	1.19	(0.41–3.47)	1
US	12	214	1.95	(0.73–5.22)	0.13
Other	12	202	2.99	(1.17–7.67)	*0.018*
B. Respiratory support					
Treatment^a^					
Non-use (ref)	20	529			
ERT	28	661	1.23	(0.61–2.47)	0.51
Disease duration^a^					
< 5 years (ref)	11	430			
5–10 years	12	319	1.12	(0.42–3.02)	1
> 10 years	25	441	2.13	(0.89–5.09)	0.051
Sex					
Male (ref)	21	420			
Female	27	770	0.67	(0.34–1.32)	0.18
Country of residence					
Netherlands (ref)	18	599			
Germany	9	189	1.28	(0.48–3.38)	1
US	13	213	2.22	(0.90–5.49)	0.10
Other	8	189	1.37	(0.52–3.62)	0.46

^a^Time-dependent covariates, updated at start of ERT; Italicized *p*-values are significant (i.e. below 0.05).

For the analysis of use of respiratory support, 1190 person-years were accumulated (median follow-up 6 years, Table 1). Over 661 treated person-years, 28 patients started using respiratory support, compared to 20 patients over 529 untreated person-years. We detected no association between ERT and the risk for starting respiratory support (HR 1.23; 95%CI 0.61–2.47). Longer disease duration again tended to increase the risk for starting respiratory support, while no significant difference was detected for sex or country of residence.

## Discussion

This is the first study to provide evidence that ERT with Alglucosidase alfa reduces the risk that adult patients with Pompe disease will become wheelchair dependent. Using data from an international cohort of almost 200 adult patients, we show that, at any point in time, a patient who received ERT had a 64% smaller probability of becoming wheelchair dependent than an untreated patient. With regard to the risk for starting respiratory support, no differences could be detected.

To patients, ambulatory and respiratory status are relevant outcomes that have a substantial impact on quality of life [6, 20]. As sometimes decades may pass between a patient’s first symptoms and their becoming ventilator or wheelchair dependent, a large cohort and long follow-up are required to study changes in these outcomes. Similarly, any study of the effect of treatment on these outcomes requires data that were obtained before and after start of this treatment. Our survey uniquely meets these requirements.

Since ambulation requires sufficient muscle strength and function, our finding that ERT reduces the risk of becoming wheelchair dependent is in line with studies reporting improvements in muscle strength and function [7–15]. With regard to our inability to demonstrate an effect of ERT on the risk for respiratory support, this may have been due to the smaller effect of ERT on respiratory muscles than on skeletal muscles, which has been reported in several earlier studies [7, 9–11, 13].

Our analyses were corrected for disease duration, country of residence and gender. Longer disease duration increased the risk for both wheelchair and respiratory support, which is in line with the progressive character of the disease, and was also concluded from earlier studies from the IPA/Erasmus MC Pompe survey and our clinical studies [21–23]. The risk of wheelchair use varied between country, potentially as a result of differences in healthcare systems or in diagnostic delays. Although the survey detected no differences between gender on the risk for wheelchair use and respiratory support, the fact that a larger proportion of men than women were already using respiratory support at inclusion in the survey suggests that there may be gender differences in lung function. This was also suggested by our earlier study in untreated adults, where the decline in lung function was faster in men [23]. More research is needed to elucidate these differences.

The IPA survey is an open cohort into which patients are continually included. It has been shown to be a good reflection of the clinical spectrum of adult patients with Pompe disease [20]. Some bias may have occurred as some patients will become lost to follow-up in such a long-term study. In addition to correcting for confounders, bias was further minimized in this study through the time dependent nature of the analysis, as the same patient could contribute to both the treated and untreated period.

The difference between being able to walk and needing a wheelchair is very tangible, and ERT’s reduction of the risk for becoming wheelchair dependent is an important improvement. Nevertheless, a proportion of treated patients still become wheelchair dependent at some point in their life. Hence, while ERT shows positive clinical effects in adult patients with Pompe disease, we also conclude that there is still room for improvement.

## Conclusion

ERT reduces the risk for wheelchair dependency in adult Pompe patients. Since ambulation provides independence, we believe this is of key importance to patients. An effect of ERT on the risk for respiratory support could not be demonstrated.

## Abbreviations

CI: Confidence Interval
ERT: Enzyme replacement therapy
HR: Hazard Ratio
IPA: International Pompe Association
